# Supplementary material for: Efficient shallow learning as an alternative to deep learning
Source: Sci Rep. 2023 Apr 20;13:5423. doi: 10.1038/s41598-023-32559-8 (PMC10119101; doi:10.1038/s41598-023-32559-8)
Supplement: Supplementary file 1 — Supplementary Information. [file 41598_2023_32559_MOESM1_ESM.pdf]

Supplementary Information

Efficient shallow learning as an alternative to deep learning

Yuval Meir[1], Ofek Tevet[1], Yarden Tzach[1], Shiri Hodassman[1], Ronit D. Gross[1] and Ido Kanter[1,2*]

[1]Department of Physics, Bar-Ilan University, Ramat-Gan, 52900, Israel.

[2]Gonda Interdisciplinary Brain Research Center, Bar-Ilan University, Ramat-Gan, 52900, Israel.

[*]Corresponding author email: ido.kanter@biu.ac.il

Generalized LeNet and VGG-16 architectures and initial weights. The generalized LeNet architecture (Fig. 1A) consists of two consecutive convolutional layers of size (5×5) and depths d_1 and d_2 and three fully connected layers of sizes $25 \times d_2$, 120, 84. After each convolutional layer, max-pooling consisting of (2×2) was applied[1]. The softmax function was applied to the ten outputs. The generalized VGG-16 architecture (Fig. 2A) consists of 16 layers[2], 13 convolutional layers with a single zero-padding around each input, and three fully connected layers with 4096 hidden units each. In the first four convolutional layers, max-pooling consisting of (2×2) was applied every two convolutions. In the rest of the nine convolutional layers, a max-pooling layer was applied after every three convolutional layers. After each convolutional layer, a batch normalization layer was applied. The softmax function was applied to the ten outputs. In both architectures, the ReLU activation function was assigned to each hidden unit, and all weights were initialized using a Gaussian distribution with a zero mean and standard deviation according to the He normal initialization[3].

Data preprocessing. Each input pixel of an image (32×32) from the CIFAR-10 database was divided by the maximal pixel value, 255, multiplied by 2, and subtracted by 1, such that its range was $[-1, 1]$. In all simulations, data augmentation was used, derived from the original images, by random horizontally flipping and translating up to four pixels in each direction.

Optimization. The cross-entropy cost function was chosen for the classification task and was minimized using the stochastic gradient descent algorithm[4,5]. The maximal accuracy was determined by searching through the hyper-parameters (see below). Cross-validation was confirmed using several validation databases, each consisting of $10{,}000$ random examples, as in the test set. The averaged results were in the same standard deviation (Std) as the reported average success rates. The Nesterov momentum[6] and L2 regularization method[7] were applied.

Hyper-parameters. The hyper-parameters η (learning rate), μ (momentum constant[6]), and α (regularization L2[7]) were optimized for offline learning, using a mini-batch size of 100 inputs selected such that each of the ten labels of the

CIFAR-10 dataset appear ten times. The learning rate decay schedule[8,9] was also optimized such that it was multiplied by the decay factor, q, every Δt epochs, and was denoted as $(q, \Delta t)$.

Figure 1					
d_1	d_2	η	μ	α	epochs
1	2 and 3	0.028	0.850	9.5e-4	240
2	5 and 6	0.028	0.850	9.5e-4	240
3	8	0.028	0.905	9.5e-4	220
6	16	0.028	0.910	9.5e-4	280
12	32	0.028	0.915	9.5e-4	240
18	48	0.028	0.950	9.5e-4	280

Table 1. Hyper-parameter used in Figure 1B.

The decay schedule for the learning rate is defined as follows:

$$(q, \Delta t) = \begin{cases} (0.8,10) & \text{epoch} \leq 120 \\ (0.7,10) & \text{epoch} > 120 \end{cases}$$

For LeNet, the conservation constant was $\frac{d_2}{d_1} = \frac{16}{6} = \frac{8}{3}$. For $d_1 = 1$ and $d_1 = 2$ the conservation law results in non-integer values $d_2 = 2\frac{2}{3}$ and $d_2 = 5\frac{1}{3}$, respectively. Assuming a power law, the following interpolation was performed for $d_1 = 1$:

$$\ln\left(\epsilon(d_2 = 2\frac{2}{3})\right) = \frac{\ln\left(2\frac{2}{3}\right) - \ln(2)}{\ln(3) - \ln(2)} \cdot \ln(\epsilon(d_2 = 2)) + \frac{\ln(3) - \ln\left(2\frac{2}{3}\right)}{\ln(3) - \ln(2)} \cdot \ln\left(\epsilon(d_2 = 3)\right)$$

and for $d_1 = 2$:

$$\ln\left(\epsilon(d_2 = 5\frac{1}{3})\right) = \frac{\ln\left(5\frac{1}{3}\right) - \ln(5)}{\ln(6) - \ln(5)} \cdot \ln(\epsilon(d_2 = 5)) + \frac{\ln(6) - \ln\left(5\frac{1}{3}\right)}{\ln(6) - \ln(5)} \cdot \ln\left(\epsilon(d_2 = 6)\right)$$

Note that the weighted arithmetic mean yields the same results (at least up to two leading digits).

Figure 2				
d	η	μ	α	epochs
8	0.01	0.920	9e-4	200
16	0.01	0.975	1.5e-3	200
32	0.01	0.965	9.5e-4	200
64	0.028	0.975	1.5e-3	200

Table 2. Hyper-parameter used in Figure 2B.

The decay schedule for the learning rate is defined as follows:

$$(q, \Delta t) = (0.6, 20)$$

Figure 4A					
Constant = $\frac{4}{3}$					
d_1	d_2	η	μ	α	epochs
3	4	0.035	0.900	1e-5	200
6	8	0.030	0.975	1e-5	200
12	16	0.030	0.965	4e-5	200
18	24	0.025	0.975	2e-4	200

Table 3. Hyper-parameter used in Figure 4A, for constant = 4/3.

The decay schedule for the learning rate for $d_1 = 3, 6$ and 12 is defined as follows:

$$(q, \Delta t) = \begin{cases} (0.9, 10) & \text{epoch} \leq 60 \\ (0.85, 10) & \text{epoch} > 60 \end{cases}$$

For $d_1 = 18$, the schedule is defined as follows:

$$(q, \Delta t) = \begin{cases} (0.95, 10) & \text{epoch} \leq 30 \\ (0.9, 10) & 30 < \text{epoch} \leq 60 \\ (0.8, 10) & 60 < \text{epoch} \leq 200 \end{cases}$$

Constant = $\frac{16}{3}$					
d_1	d_2	η	μ	α	epochs
3	16	0.028	0.940	9e-4	200
6	32	0.006	0.975	9e-4	200
12	64	0.010	0.975	9e-4	200
18	96	0.010	0.975	1.5e-3	200

Table 4. Hyper-parameter used in Figure 4A, for constant = 16/3.

The decay schedule for the learning rate for $d_1 = 3$ is defined as follows:

$$(q, \Delta t) = \begin{cases} (0.8,10) & \text{epoch} \le 120 \\ (0.7,10) & \text{epoch} > 120 \end{cases}$$

For $d_1 = 6, 12,$ and 18, the schedule is defined as follows:

$$(q, \Delta t) = (0.6,20)$$

**Figure 4B**				
Constant = $\frac{3}{2}$				
d	η	μ	α	epochs
16	0.008	0.975	9e-4	200
32	0.007	0.975	1.5e-3	200
64	0.002	0.970	3e-3	200
Constant = $\frac{5}{2}$				
d	η	μ	α	epochs
16	0.010	0.975	9e-4	200
32	0.010	0.965	9e-4	200
64	0.015	0.975	9e-4	200

Table 5. Hyper-parameter used in Figure 4B, for constants 3/2 and 5/2.

The decay schedule for the learning rate is defined as follows:

$$(q, \Delta t) = (0.6,20)$$

Generalization error (ϵ)**.** The evaluation of ϵ is performed by testing the network on 10000 test examples, that were not used in the training. The network's decision was taken as the output unit with the maximum value among the ten output units.

Raw data of the graphs. The raw data for the four graphs are presented in the following tables.

Figure 1			
d_1	d_2	ϵ	Std
1	2	0.524	0.0105
1	3	0.479	0.0151
1	2 and 3	0.511	0.0128
2	5	0.394	0.0086
2	6	0.375	0.0064
2	5 and 6	0.382	0.0075
3	8	0.320	0.0083
6	16	0.230	0.0042
12	32	0.181	0.0023
18	48	0.158	0.0031

Table 6. Raw data for Figure 1B.

Figure 2		
d	ϵ	Std
8	0.1496	0.0027
16	0.1097	0.0040
32	0.08334	0.0011
64	0.0644	0.0019

Table 7. Raw data for Figure 2B.

Figure 3A (LeNet)	
d_1	MAdds
6	651.72K
19	3.7M
44	15.82M
86	54.99M
164	190M
**Figure 3A (VGG-16)**	
d	MAdds
4	18.28M
8	22.17M
16	37.25M
32	96.61M
64	332M

Table 8. Raw data for Figure 3A.

Figure 3B		
ϵ	GMAdd (LeNet)	GMAdd (VGG-16)
0.0481	0.63	1.27
0.0180	74	163
0.0095	1660	3860
0.0050	37640	92480

Table 9. Raw data for Figure 3B.

Figure 3C	
ϵ	Complexity ratio [LeNet / VGG-16]
0.2306	0.036
0.1814	0.093
0.1580	0.160
0.1010	0.412
0.0793	0.487
0.0597	0.507
0.0449	0.498
0.0350	0.485
0.0059	0.413

Table 10. Raw data for Figure 3C.

Figure 4A		
Constant = $\frac{4}{3}$		
d_1	ϵ	Std
3	0.388	0.0108
6	0.291	0.0140
12	0.222	0.0058
18	0.187	0.0036
Constant = $\frac{16}{3}$		
d_1	ϵ	Std
3	0.277	0.0029
6	0.207	0.0043
12	0.167	0.0013
18	0.146	0.0010

Table 11. Raw data for Figure 4A, for constants 4/3 and 16/3.

Figure 4B		
Constant = $\frac{3}{2}$		
d	ϵ	Std
16	0.129	0.0012
32	0.096	0.0022
64	0.074	0.0009
Constant = $\frac{5}{2}$		
d	ϵ	Std
16	0.094	0.0022
32	0.073	0.0011
64	0.060	0.0008

Table 12. Raw data for Figure 4B, for constants 3/2 and 5/2.

Enhanced VGG-16. The enhanced architecture extends the number of filters following the conservation law to the fifth convolution set (Fig. 2A). At convolutional layers 11-13 the number of filters is extended to $16d$ according to the conservation law, instead of $8d$ in the original architecture. This extended architecture was tested on an initial filter size of $d = 16$, resulting in 256 filters for the fifth convolution set and yielding an average error rate of 0.0937, an improvement from the original VGG-16 (0.11).

Learning in enhanced VGG-16				
d_1	η	μ	α	epochs
16	0.007	0.975	2.0e-3	200

Table 13. Hyper-parameters for enhanced VGG-16.

The decay schedule for the learning rate is defined as follows:

$$(q, t) = (0.6, 20)$$

Multi-Adds calculations. The complexity of each network was calculated based on the number of multiplication-add operations performed [10] in the forward and BP of a single input image.

Statistics. Statistics for all simulations were obtained using at least 10 samples.

1. le Cun, Y. *et al.* Handwritten digit recognition: applications of neural network chips and automatic learning. *IEEE Communications Magazine* **27**, 41–46 (1989).

2. Simonyan, K. & Zisserman, A. Very Deep Convolutional Networks for Large-Scale Image Recognition. (2014).

3. He, K., Zhang, X., Ren, S. & Sun, J. Delving Deep into Rectifiers: Surpassing Human-Level Performance on ImageNet Classification. in *2015 IEEE International Conference on Computer Vision (ICCV)* 1026–1034 (IEEE, 2015). doi:10.1109/ICCV.2015.123.

4. Robbins, H. & Monro, S. A Stochastic Approximation Method. *The Annals of Mathematical Statistics* **22**, 400–407 (1951).

5. Kiefer, J. & Wolfowitz, J. Stochastic Estimation of the Maximum of a Regression Function. *The Annals of Mathematical Statistics* **23**, 462–466 (1952).

6. Botev, A., Lever, G. & Barber, D. Nesterov's accelerated gradient and momentum as approximations to regularised update descent. in *2017 International Joint Conference on Neural Networks (IJCNN)* 1899–1903 (IEEE, 2017). doi:10.1109/IJCNN.2017.7966082.

7. Schmidhuber, J. Deep learning in neural networks: An overview. *Neural Networks* **61**, 85–117 (2015).

8. He, K., Zhang, X., Ren, S. & Sun, J. Deep Residual Learning for Image Recognition. (2015).

9. You, K., Long, M., Wang, J. & Jordan, M. I. How Does Learning Rate Decay Help Modern Neural Networks? (2019).

10. Sandler, M., Howard, A., Zhu, M., Zhmoginov, A. & Chen, L.-C. MobileNetV2: Inverted Residuals and Linear Bottlenecks. (2018).
